# Cutis Tricolor‐Like Pigmentary Mosaicism in Mowat–Wilson Syndrome: Phenotypic Overlap With Ruggieri–Happle Syndrome

**DOI:** 10.1111/pde.70294

**Published:** 2026-06-14

**Authors:** Didier Bessis, Pierre Meyer, Marjolaine Willems

**Affiliations:** ^1^ Department of Dermatology University of Montpellier, Reference Centre for Rare Skin Diseases (MAGEC‐Sud), Saint‐Eloi Hospital, CHU Montpellier Montpellier France; ^2^ INSERM U1058 Montpellier France; ^3^ Department of Paediatric Neurology University of Montpellier, CHU Montpellier Montpellier France; ^4^ Department of Medical Genetics University of Montpellier, Reference Center for Rare Diseases, Developmental Disorders and Multiple Congenital Anomalies, Arnaud de Villeneuve Hospital, CHU Montpellier Montpellier France

**Keywords:** cutis tricolor, Mowat–Wilson syndrome, Ruggieri–Happle syndrome, ZEB2

## Abstract

Cutis tricolor (CT) is a rare pigmentary mosaicism characterized by the coexistence of hyperpigmented and hypopigmented areas on a background of normal skin; its syndromic form, Ruggieri–Happle syndrome (RHS), is associated with neurodevelopmental delay, facial dysmorphism, skeletal abnormalities, and other systemic defects. We report a 16‐year‐old girl with molecularly confirmed Mowat–Wilson syndrome caused by a de novo pathogenic *ZEB2* frameshift variant, who presented with extensive CT‐like pigmentary mosaicism and marked phenotypic overlap with RHS. Brain MRI disclosed bilateral microphthalmia, optic nerve hypoplasia, coloboma, a shortened corpus callosum, a brainstem segmentation defect, and inferior vermian hypoplasia. This observation expands the cutaneous phenotype of *ZEB2*‐related disease and suggests possible shared developmental pathways between Mowat–Wilson syndrome and RHS.

To the Editors,

1

Cutis tricolor (CT) is a rare pigmentary mosaicism characterized by the coexistence of hyperpigmented and hypopigmented areas on a background of normal skin, arranged in patches, linear streaks, or spiral patterns [1]. Two phenotypic subsets have been delineated: a purely cutaneous form and a complex neurocutaneous phenotype termed Ruggieri–Happle syndrome (RHS), in which CT is associated with facial dysmorphism, neurodevelopmental delay, skeletal anomalies, and systemic defects (Table S1) [2]. The molecular basis of RHS remains unknown. Here, we report a patient with extensive CT‐like pigmentary mosaicism and marked phenotypic overlap with RHS, in whom a heterozygous pathogenic *ZEB2* variant was identified.

A 16‐year‐old girl was born at term to healthy, nonconsanguineous Algerian parents after an uneventful pregnancy. At birth, she had bilateral microphthalmia, right‐sided sclerocornea, and pigmentary skin abnormalities. She had severe global developmental delay and did not acquire independent walking or speech. Examination disclosed proportionate growth retardation (height, −3 SD; weight, −2 SD), microcephaly (−2 SD), spastic tetraparesis, and blindness. Facial features included enophthalmia, laterally flared eyebrows with medial sparing, hypertelorism, a deep nasal bridge, a large bulbous nose with wide nares, uplifted earlobes, a prominent columella, a short broad philtrum, thick lips, and a prominent chin (Figure 1). Cutaneous examination showed two large hyperpigmented patches with regular yet scalloped borders contiguous with hypopigmented skin. One extended from the left thorax to the posterior aspect of the left upper limb, and the other involved the waist and abdomen (Figure 2A,B). Brain MRI performed at 6 years of age revealed bilateral microphthalmia, optic nerve hypoplasia, coloboma, a shortened corpus callosum, a brainstem segmentation defect, and inferior vermian hypoplasia. Karyotype and chromosomal microarray analyses performed on blood and hypopigmented skin were normal. Because the association of severe global developmental delay, microcephaly, congenital ocular anomalies, brain malformations, and a characteristic facial gestalt was clinically suggestive of Mowat–Wilson syndrome (MWS), targeted Sanger sequencing of ZEB2 was performed. This analysis identified a de novo heterozygous pathogenic frameshift variant, NM_014795.5:c.515_516delCA; p.(Thr172Serfs*9), thereby establishing the molecular diagnosis of MWS. Subsequent exome sequencing did not identify any additional pathogenic variant.

**FIGURE 1:**
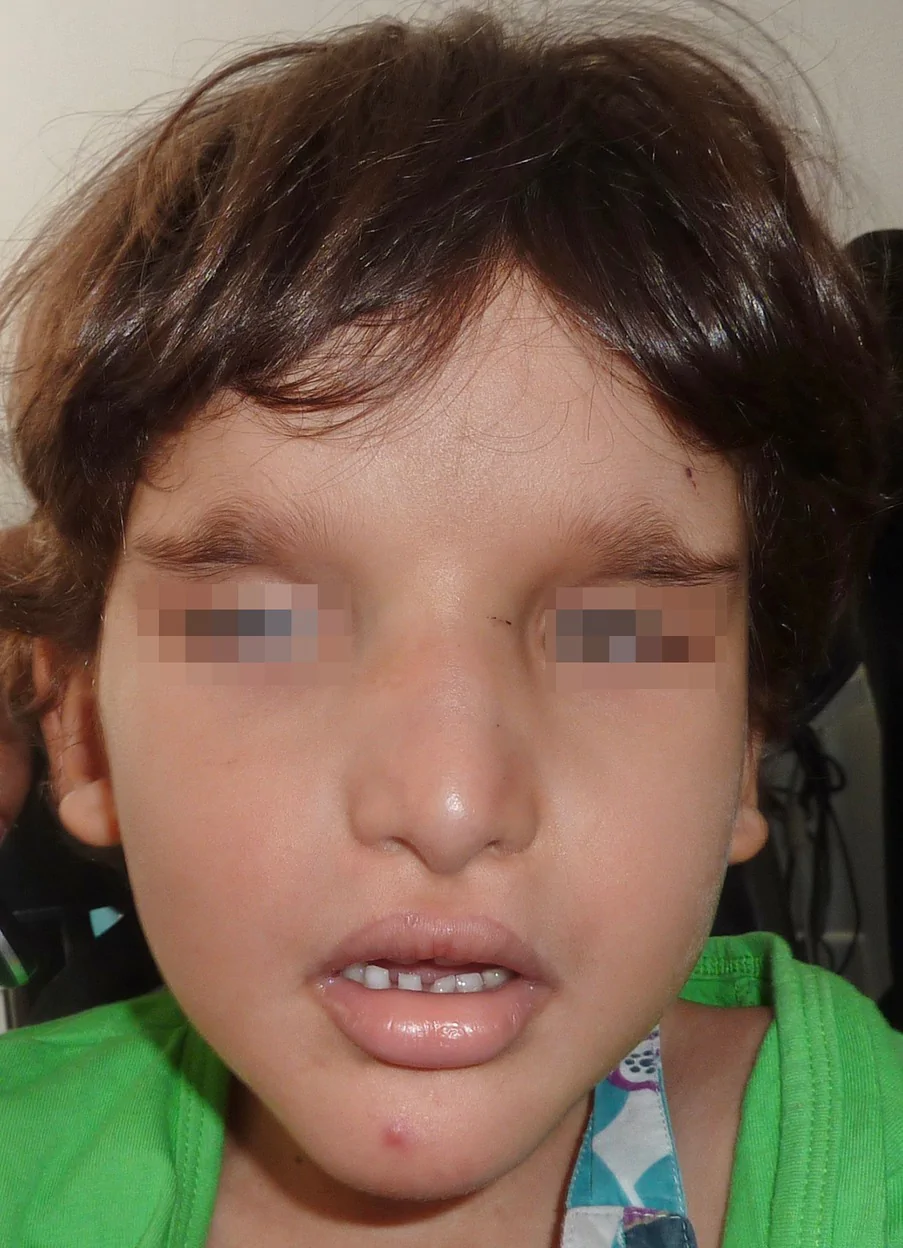
Facial features at 6 years of age, consistent with Mowat–Wilson syndrome. Laterally flared eyebrows with medial sparing, hypertelorism, deep nasal bridge, large bulbous nose with wide nares, uplifted earlobes, prominent columella, short broad philtrum, thick lips, and prominent chin.

**FIGURE 2:**
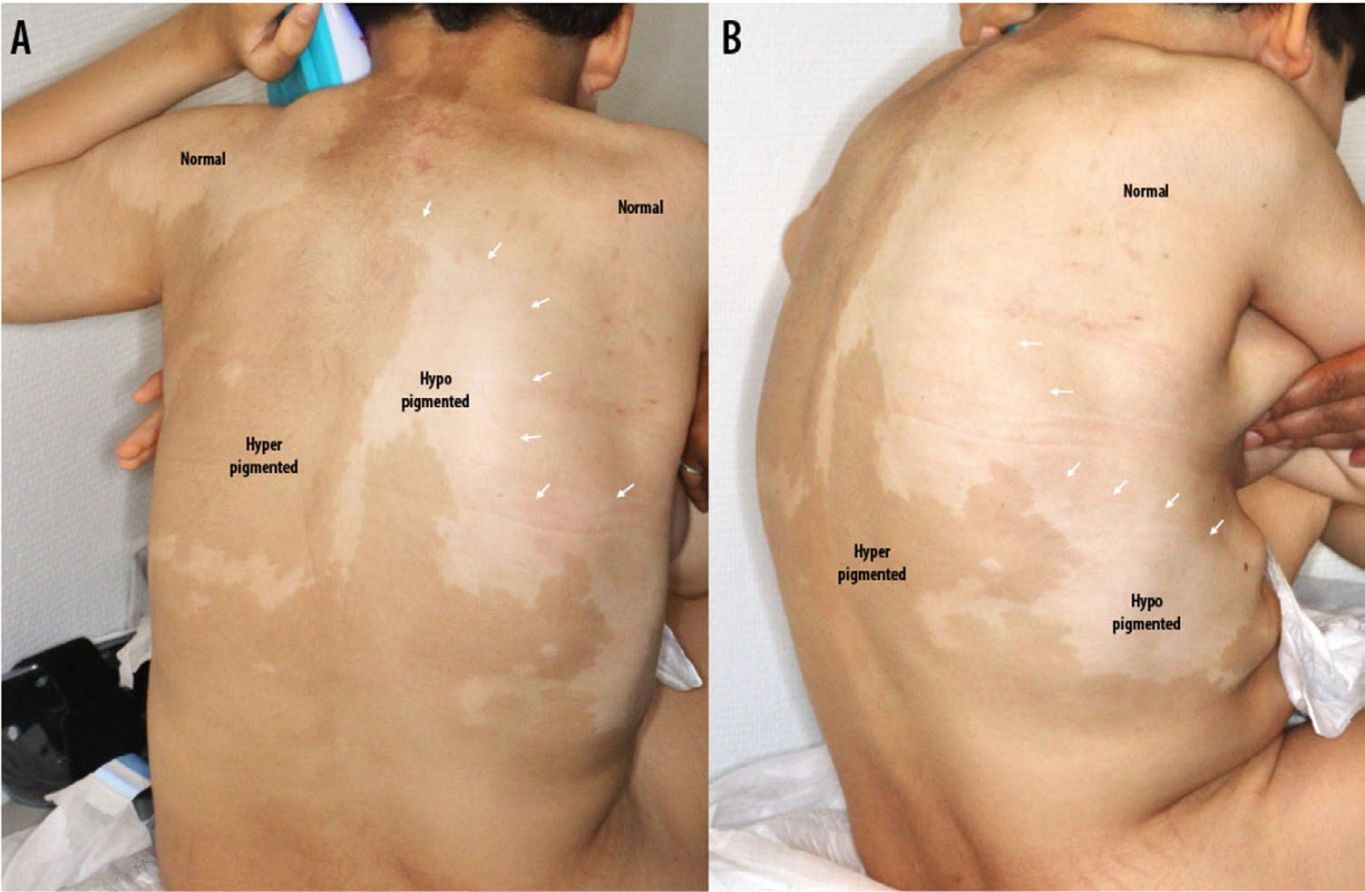
Cutis tricolor‐like pigmentary mosaicism of the trunk at 16 years of age. (A) Posterior view showing a sash‐like distribution of large oblique hypopigmented patches in close apposition to darker, clinically hyperpigmented areas, interspersed with islands of clinically normal skin. White arrows indicate the junction between normal and hypopigmented skin. (B) Lateral view better illustrating the tripartite pattern, with normally pigmented skin visible on the shoulder convexity and at the root of the right arm, a large hypopigmented patch on the flank, and adjacent darker hyperpigmented areas along the lateral and the lower borders. White arrows again indicate the normal–hypopigmented interface. The pattern does not fit conventional categories of pigmentary mosaicism, such as Blaschkoid, checkerboard, linear, or phylloid patterns.

This observation documents a patient with molecularly confirmed MWS who also exhibited a CT‐like pigmentary pattern and substantial phenotypic overlap with RHS. In published MWS cohorts, cutaneous manifestations are usually reported briefly and nonspecifically, including “hyperpigmentation” and/or “hypopigmentation” in approximately 15% [3, 4, 5]. By contrast, our patient showed a distinctive CT‐like pigmentary pattern, not fitting classic categories of pigmentary mosaicism, and resembling the “unclassifiable” configuration repeatedly emphasized in RHS reports [2]. The phenotypic overlap between RHS and MWS extends beyond pigmentation, particularly with respect to facial dysmorphism, neurodevelopmental impairment, and structural brain anomalies. In our patient, however, the presence of microcephaly and microphthalmia, together with a de novo pathogenic *ZEB2* variant, clearly supports MWS as the primary diagnosis. Although this observation raises the possibility that ZEB2‐related disease may be associated with a CT‐like phenotype, a single case is insufficient to consider RHS and MWS as allelic conditions or to establish ZEB2 variants as a cause of RHS. Rather than supporting nosological unification, this case expands the pigmentary phenotype of *ZEB2*‐related disease and suggests shared developmental pathways between MWS and RHS. Consistent with this cautious interpretation, Ruggieri et al. found no *ZEB2* abnormality by FISH and sequencing in 14 of 19 patients with RHS [2], suggesting phenotypic convergence, genetic heterogeneity, or both. Such overlap remains biologically plausible, as ZEB2 is a key regulator of neural crest development and may influence melanocyte differentiation through MITF (microphthalmia‐associated transcription factor).

## Author Contributions

**Didier Bessis:** writing, visualization, conceptualization. **Pierre Meyer and Marjolaine Willems:** writing – review.

## Funding

The authors have nothing to report.

## Ethics Statement

The authors have nothing to report.

## Consent

Written informed consent for publication was obtained from the patient's legal guardians.

## Conflicts of Interest

The authors declare no conflicts of interest.

## Supporting information


**Table S1:** Reported patients with cutis tricolor syndrome or Ruggieri–Happle syndrome.

## Data Availability

The data that support the findings of this study are available from the corresponding author upon reasonable request.
