# Supplementary material for: Cutis Tricolor‐Like Pigmentary Mosaicism in Mowat–Wilson Syndrome: Phenotypic Overlap With Ruggieri–Happle Syndrome
Source: Pediatr Dermatol. 2026 Jun 14;43(5):1247–9. doi: 10.1111/pde.70294 (PMC13617161; doi:10.1111/pde.70294)
Supplement: Supplementary file 1 [file PDE-43-1247-s001.docx]

| **Table 1. Reported patients with cutis tricolor syndrome or Ruggieri-Happle syndrome** | | | | | | | | | | |  | |  |  |  |
| --- | --- | --- | --- | --- | --- | --- | --- | --- | --- | --- | --- | --- | --- | --- | --- |
| **Reference** | **Case** | **Sex** | **Age (y)** | **Cutis tricolor** | **Cutis tricolor** | **Facial morphological abnormalities** | **Neurological manifestations** | **Neuroimaging features** | **EEG abnormalities** | **Ocular manifestations** | | **Osteoarticular manifestations** | **Other manifestations** | **Genetic analysis** | |
|  |  |  |  |  |  |  |  |  |  |  | |  |  |  |  |
| Happle et al., 1997 (J Med Genet) | 1 (case also reported by Lionetti et al, 2010) | M | 28 | Macules (multiple) | . Face (+/-)  . Upper limb (right)  . Neck (right) | . Coarse face  . Orbital bossing  . Brushy eyebrows  . Epicanthus  . Deep nasal bridge  . Low set/angulated ears  . Large/bulbous nose  . Broad nostrils  . Wide philtrum  . Prognathism | . Cognitive delay (severe)  . Delayed language  . Epilepsy (generalised tonic clonic seizures)  . Sensorineural deafness  . Mental retardation (severe) | No | Yes | No cataract | | . Short stature  . Short neck  . Scoliosis  . Kyphosis  . Lordosis  . Pectus excavatum  . Skull dysplasia  . Vertebral dysplasia  . Long bone dysplasia | Ventricular septal defect | . No pathogenic variant of *ZEB2* (*ZFHX1B*) gene  . Karyotyping (standard and high resolution) normal |  |
| . Ruggieri et al., 2000 (Eur J Pediatr)  . Ruggieri et al., 2003 (Am J Med Genet)  . Ruggieri et al., 2009 (Br J Ophtalmol)  . Lionetti et al., 2010 (Neuropediatrics)  . Ruggieri et al., 2011 (Acta Paediatr) | 2 | F | 2 | Patches | . Trunk  . Lower limbs | . Coarse face  . Facial asymmetry  . Frontal bossing  . Brushy eyebrows  . Hypertelorism  . Deep nasal bridge  . Large/bulbous nose  . Large nostrils  . Low set/angulated ears  . Wide philtrum  . Thick lips  . Prominent chin  . Curly hair | . Cognitive delay (severe)  . Delayed language  . Epilepsy (partial seizure type, drug resistant)  . Hypotonia  . Poor coordination  . Mental retardation (severe)  . Behavioural disturbances | White matter anomalies (brain) | Yes | No cataract | | . Scoliosis  . Skull dysplasia  . Vertebral dysplasia  . Long bone dysplasia | No | . No pathogenic variant of *ZEB2* (*ZFHX1B*) gene  . Karyotyping (standard and high resolution) normal |  |
|  | 3 | M | 2 | . Patches  . Linear | . Trunk | No | . Cognitive delay (mild)  . Delayed language  . Muscular weakness  . Mental retardation (mild) | No | No | No cataract | | . Scoliosis  . Kyphosis | No | . No pathogenic variant of *ZEB2* (*ZFHX1B*) gene  . Karyotyping (standard and high resolution) normal |  |
|  | 4 | F | 5 | Macules (multiple) | . Trunk  . Lower limb (right) | No | . Cognitive delay (moderate)  . Delayed language  . Behavioural disturbances  . Poor coordination  . Muscular weakness  . Mental retardation (moderate) | No | No | . No cataract  . Nystagmus | | . Skull dysplasia  . Vertebral dysplasia  . Long bone dysplasia | No | . No pathogenic variant of *ZEB2* (*ZFHX1B*) gene  . Karyotyping (standard and high resolution) normal |  |
|  | 5 | M | 8 | . Patches  . Linear | . Face  . Trunk  . Lower limbs | . Coarse face  . Facial asymmetry  . Dolichocephalism  . Frontal bossing  . Orbital bossing  . Brushy eyebrows  . Hypertelorism  . Epicanthus  . Deep nasal bridge  . Large/bulbous nose  . Large nostrils  . Low set/angulated ears  . Wide philtrum  . Thick lips  . Prominent chin | . Cognitive delay (moderate)  . Delayed language  . Behavioural disturbances  . Poor coordination  . Muscular weakness  . Walking imbalance  . Mental retardation (moderate) | . White matter anomalies (brain)  . Asymmetric ventricles | Yes | No cataract | | . Scoliosis  . Kyphosis  . Lordosis  . Short neck  . Clinodactyly  . Pectus excavatum  . Skull dysplasia  . Vertebral dysplasia  . Long bone dysplasia | No | . No pathogenic variant of *ZEB2* (*ZFHX1B*) gene  . Karyotyping (standard and high resolution) normal |  |

| **Reference** | **Case** | **Sex** | **Age (y)** | **Cutis tricolor** | **Cutis tricolor** | **Facial morphological abnormalities** | **Neurological manifestations** | **Neuroimaging features** | **EEG abnormalities** | **Ocular manifestations** | **Osteoarticular manifestations** | **Other manifestations** | **Genetic analysis** | |
| --- | --- | --- | --- | --- | --- | --- | --- | --- | --- | --- | --- | --- | --- | --- |
|  | 6 | F | 10 | Patches | . Trunk  . Upper limbs  . Lower limbs | Brushy eyebrows | No | No | No | No cataract | No | No | . No pathogenic variant of *ZEB2* (*ZFHX1B*) gene  . Karyotyping (standard and high resolution) normal |  |
|  | 7 | M | 11 | Sash-like (Spiral) | Trunk | . Coarse face  . Facial asymmetry  . Frontal bossing  . Brushy eyebrows  . Epicanthus  . Deep nasal bridge  . Large/bulbous nose  . Large nostrils  . Wide philtrum  . Thick lips | . Cognitive delay (moderate)  . Delayed language  . Poor coordination  . Hypotonia  . Behavioural disturbances  . Epilepsy (partial seizure type)  . Mental retardation (moderate) | No | Yes | No cataract | . Skull dysplasia  . Vertebral dysplasia  . Long bone dysplasia | No | . No pathogenic variant of *ZEB2* (*ZFHX1B*) gene  . Karyotyping (standard and high resolution) normal |  |
|  | 8 | F | 14 | . Patches  . Linear | . Trunk  . Upper limbs  . Lower limbs | . Coarse face  . Facial asymmetry  . Dolichocephalism  . Frontal bossing  . Orbital bossing  . Brushy eyebrows  . Hypertelorism  . Deep nasal bridge  . Large/bulbous nose  . Wide philtrum  . Prominent chin | . Cognitive delay (mild)  . Delayed language  . Muscular weakness (+/-)  . Nystagmus  . Walking imbalance  . Mental retardation (mild) | Asymmetric ventricles | Yes | No cataract | . Scoliosis  . Kyphosis  . Lordosis  . Short neck  . Clinodactyly  . Pectus excavatum  . Skull dysplasia  . Vertebral dysplasia  . Long bone dysplasia | No | . No pathogenic variant of *ZEB2* (*ZFHX1B*) gene  . Karyotyping (standard and high resolution) normal |  |
|  | 9 (case 2 from Ruggieri et al, 2000 and, case 1 from Ruggieri et al, 2009) | M | 14 | Patches | . Face  . Trunk  . Lower limbs | . Coarse face  . Facial asymmetry  . Dolichocephalism  . Frontal bossing  . Orbital bossing  . Brushy eyebrows  . Hypertelorism  . Epicanthus  . Deep nasal bridge  . Large/bulbous nose  . Large nostrils  . Low set/angulated ears  . Wide philtrum  . Thick lips  . Prominent chin | . Cognitive delay (moderate)  . Delayed language  . Muscular weakness  . Epilepsy (generalised tonic clonic seizures, drug resistant)  . Hearing defect  . Mental retardation (moderate) | White matter anomalies (brain, subcortical and periventricular) | No | Cataract | . Scoliosis  . Kyphosis  . Lordosis  . Short neck  . Pectus excavatum  . Skull dysplasia  . Vertebral dysplasia  . Long bone dysplasia | No | . No pathogenic variant of *ZEB2* (*ZFHX1B*) gene  . Karyotyping (standard and high resolution) normal |  |
|  | 10 | M | 14 | Patches | . Trunk  . Upper limbs  . Lower limbs | . Coarse face  . Brushy eyebrows | No | White matter anomalies (brain) | Yes | No cataract | . Scoliosis  . Kyphosis  . Pectus excavatum | No | . No pathogenic variant of *ZEB2* (*ZFHX1B*) gene  . Karyotyping (standard and high resolution) normal |  |

| **Reference** | **Case** | **Sex** | **Age (y)** | **Cutis tricolor** | **Cutis tricolor** | **Facial morphological abnormalities** | **Neurological manifestations** | **Neuroimaging features** | **EEG abnormalities** | **Ocular manifestations** | **Osteoarticular manifestations** | **Other manifestations** | **Genetic analysis** | |
| --- | --- | --- | --- | --- | --- | --- | --- | --- | --- | --- | --- | --- | --- | --- |
|  | 11 (case from Ruggieri et al, 2003 and case 3 from Ruggieri et al, 2009) | F | 15 | . Patches  . Linear | . Trunk  . Upper limbs | . Coarse face  . Facial asymmetry  . Dolichocephalism  . Frontal bossing  . Orbital bossing  . Brushy eyebrows  . Hypertelorism  . Epicanthus  . Deep nasal bridge  . Large/bulbous nose  . Large nostrils  . Low set/angulated ears  . Wide philtrum  . Thick lips  . Prominent chin | . Cognitive delay (moderate)  . Delayed language  . Behavioural disturbances  . Poor coordination  . Muscular weakness  . Epilepsy (generalised tonic clonic seizures)  . Mental retardation (moderate/severe) | Corpus callosum anomalies | Yes | Cataract | . Scoliosis  . Short neck  . Clinodactyly  . Pectus excavatum  . Vertebral dysplasia | No | . No pathogenic variant of *ZEB2* (*ZFHX1B*) gene  . Karyotyping (standard and high resolution) normal |  |
|  | 12 | M | 17 | Sash-like (Spiral) | . Face (+/-)  . Trunk | No | No | No | No | No cataract | No | No | . No pathogenic variant of *ZEB2* (*ZFHX1B*) gene  . Karyotyping (standard and high resolution) normal |  |
|  | 13 (case 1 from Ruggieri et al, 2000 ; case 2 from Ruggieri et al, 2009) | M | 18 | Sash-like (Spiral) | . Face (+/-)  . Trunk | No | No | No | No | Cataract | . Scoliosis (idiopathic, mild type)  . Lordosis (idiopathic, mild type) | No | . No pathogenic variant of *ZEB2* (*ZFHX1B*) gene  . Karyotyping (standard and high resolution) normal |  |
|  | 14 | F | 22 | Macules (multiple) | Trunk | . Brushy eyebrows  . Thick lips  . Prominent chin | No | No | No | No cataract | . Pectus excavatum  . Skull dysplasia  . Vertebral dysplasia  . Long bone dysplasia | No | . No pathogenic variant of *ZEB2* (*ZFHX1B*) gene  . Karyotyping (standard and high resolution) normal |  |
| Ruggieri et al., 2016 (Quant Imaging Med Surg) | 15 | M | 2 | . Patches  . Linear | . Face  . Trunk  . Lower limbs | . Coarse face  . Facial asymmetry  . Dolichocephalism  . Frontal bossing  . Orbital bossing  . Brushy eyebrows  . Hypertelorism  . Epicanthus  . Deep nasal bridge  . Large/bulbous nose  . Large nostrils  . Low set/angulated ears  . Wide philtrum  . Thick lips  . Prominent chin | . Delayed developmental milestones  . Hypotonia  . Poor coordination  . Delayed language  . Behavioural anomalies  . Mental retardation (moderate) | . Asymmetric ventricles  . White matter anomalies (brain)  . Corpus callosum anomalies | Yes | ND | . Short neck  . Clinodactyly  . Scoliosis  . Pectus excavatum  . Skull dysplasia  . Vertebral dysplasia  . Long bone dysplasia | No | No array-CGH anomalies |  |

| **Reference** | **Case** | **Sex** | **Age (y)** | **Cutis tricolor** | **Cutis tricolor** | **Facial morphological abnormalities** | **Neurological manifestations** | **Neuroimaging features** | **EEG abnormalities** | **Ocular manifestations** | **Osteoarticular manifestations** | **Other manifestations** | **Genetic analysis** | |
| --- | --- | --- | --- | --- | --- | --- | --- | --- | --- | --- | --- | --- | --- | --- |
|  | 16 | F | 4 | Patches | . Trunk  . Upper limbs  . Lower limbs | . Brushy eyebrows  . Prominent chin | No | No | No | ND | . Short neck  . Clinodactyly  . Pectus excavatum | No | No array-CGH anomalies |  |
|  | 17 | F | 12 | . Patches  . Linear | . Trunk  . Upper limbs  . Lower limbs | . Coarse face  . Facial asymmetry  . Frontal bossing  . Orbital bossing  . Brushy eyebrows  . Hypertelorism  . Deep nasal bridge  . Large/bulbous nose  . Wide philtrum  . Prominent chin | . Delayed developmental milestones  . Hypotonia  . Delayed language  . Mental retardation (mild) | . Asymmetric ventricles  . Corpus callosum anomalies | Yes | Cataract | . Short neck  . Clinodactyly  . Scoliosis  . Pectus excavatum  . Skull dysplasia  . Vertebral dysplasia  . Long bone dysplasia | No | No array-CGH anomalies |  |
|  | 18 | M | 13 | Patches | . Face  . Trunk  . Lower limbs | . Coarse face  . Facial asymmetry  . Dolichocephalism  . Frontal bossing  . Orbital bossing  . Brushy eyebrows  . Hypertelorism  . Epicanthus  . Deep nasal bridge  . Large/bulbous nose  . Large nostrils  . Low set/angulated ears  . Wide philtrum  . Thick lips  . Prominent chin | . Delayed developmental milestones  . Hypotonia  . Delayed language  . Epilepsy (generalised tonic clonic seizures)  . Mental retardation (moderate) | . White matter anomalies (brain)  . Corpus callosum anomalies | No | ND | . Short neck  . Scoliosis  . Pectus excavatum  . Skull dysplasia  . Vertebral dysplasia  . Long bone dysplasia | No | No array-CGH anomalies |  |
|  | 19 | M | 14 | Macules (multiple) | Trunk | Brushy eyebrows | . Epilepsy (generalised tonic clonic seizures)  . Behavioural anomalies  . Mental retardation (mild) | No | No | ND | . Pectus excavatum | Ventricular septal defect | No array-CGH anomalies |  |

**References**

. Happle R, Barbi G, Eckert D, Kennerknecht I.["Cutis tricolor": congenital hyper- and hypopigmented macules associated with a sporadic multisystem birth defect: an unusual example of twin spotting?](https://pubmed.ncbi.nlm.nih.gov/9279762/) J Med Genet. 1997;34:676-8.

. Ruggieri M. [Cutis tricolor: congenital hyper- and hypopigmented lesions in a background of normal skin with and without associated systemic features: further expansion of the phenotype.](https://pubmed.ncbi.nlm.nih.gov/11039129/) Eur J Pediatr. 2000;159:745-9.

. Ruggieri M, Iannetti P, Pavone L. [Delineation of a newly recognized neurocutaneous malformation syndrome with "cutis tricolor".](https://pubmed.ncbi.nlm.nih.gov/12794702/) Am J Med Genet A. 2003;120A:110-6.

. Ruggieri M, Iannetti F, Polizzi A, et al. [Cataracts in three children with a newly recognised neurocutaneous malformation phenotype with "cutis tricolor".](https://pubmed.ncbi.nlm.nih.gov/19098051/) Br J Ophthalmol. 2009;93:127-8.

. [Lionetti E, Pavone P, Kennerknecht I et al. Neurological manifestations in individuals with pure cutaneous or syndromic (Ruggieri-Happle syndrome) phenotypes with « Cutis Tricolor » : a study of 14 cases.](https://pubmed.ncbi.nlm.nih.gov/32424176/) Neuropediatrics. 2010;41:60-65.

. Ruggieri M, Roggini M, Kennerknecht I, et al. [Spectrum of skeletal abnormalities in a complex malformation syndrome with "cutis tricolor" (Ruggieri-Happle syndrome).](https://pubmed.ncbi.nlm.nih.gov/21143296/) Acta Paediatr. 2011;100:121-7.

. Ruggieri M, Polizzi A, Schepis C, et al P. [Cutis tricolor: a literature review and report of five new cases.](https://pubmed.ncbi.nlm.nih.gov/27942472/) Quant Imaging Med Surg. 2016;6:525-534.
